# Palatal Tremor – Pathophysiology, Clinical Features, Investigations, Management and Future Challenges

**DOI:** 10.5334/tohm.188

**Published:** 2020-10-08

**Authors:** Shakya Bhattacharjee

**Affiliations:** 1 Neurology, Plymouth Hospital NHS Trust, UK

**Keywords:** palatal, tremor, hypertrophic, olivary, degeneration

## Abstract

**Background:**

Palatal tremor is involuntary, rhythmic and oscillatory movement of the soft palate. Palatal tremor can be classified into three subtypes; essential, symptomatic and palatal tremor associated with progressive ataxia.

**Methods:**

A thorough Pubmed search was conducted to look for the original articles, reviews, letters to editor, case reports, and teaching neuroimages, with the keywords “essential”, “symptomatic palatal tremor”, “myoclonus”, “ataxia”, “hypertrophic”, “olivary” and “degeneration”.

**Results:**

Essential palatal tremor is due to contraction of the tensor veli palatini muscle, supplied by the 5^th^ cranial nerve. Symptomatic palatal tremor occurs due to the contraction of the levator veli palatini muscle, supplied by the 9%^th^ and 10%^th^ cranial nerves. Essential palatal tremor is idiopathic, while symptomatic palatal tremor occurs due to infarction, bleed or tumor within the Guillain-Mollaret triangle. Progressive ataxia and palatal tremor can be familial or idiopathic. Symptomatic palatal tremor and sporadic progressive ataxia with palatal tremor show signal changes in inferior olive of medulla in magnetic resonance imaging. The treatment options available for essential palatal tremor are clonazepam, lamotrigine, sodium valproate, flunarizine and botulinum toxin. The treatment of symptomatic palatal tremor involves the treatment of the underlying cause.

**Discussion:**

Further studies are required to understand the cause and pathophysiology of Essential palatal tremor and progressive ataxia and palatal tremor. Similarly, the link between tauopathy and palatal tremor associated progressive ataxia needs to be explored further. Oscillopsia and progressive ataxia are more debilitating than palatal tremor and needs new treatment approaches.

## Introduction

A tremor is an involuntary, rhythmic and oscillatory movement of one or more body parts at a fixed amplitude and frequency [1,2,3]. A palatal tremor (PT) is a rare type of tremor involving the soft palate. It can be unilateral or bilateral [1,2]. Previously, palatal tremor was known as palatal myoclonus but it was subsequently renamed ‘palatal tremor’ during the First International Congress of Movement Disorders to acknowledge the continuous and rhythmic nature of the palatal movement (although it should be noted that segmental myoclonus can also be rhythmic). Palatal tremors do not oscillate around a point, unlike any other type of tremor. Palatal tremors can have huge variability in frequency (20–420 cycles/in), unlike other commonly-described tremors [3]. Despite these controversies, ‘palatal tremor’ is the most widely accepted term.

Two types of palatal tremor are described in the literature: essential palatal tremor (EPT) and symptomatic palatal tremor (SPT). EPT reveals no underlying structural pathology [3,4]. SPT happens due to any lesion within the dentato-rubral-olivary pathway [1,3]. Progressive ataxia and palatal tremor (PAPT) is traditionally considered to be a special subtype of SPT where the ataxia worsens independent of the tremor [1,3]. Each subtype of palatal tremor has different aetiologies, clinical features and prognosis.

## Methods

A thorough Pubmed search was conducted to look for original articles, reviews, letters to the editor, case reports and teaching neuroimages of relevance. The keywords used were ‘essential”, ‘’symptomatic”, “palatal”, “tremor”, “myoclonus”, “ataxia”,’’ hypertrophic”, “olivary” and “degeneration.”

## Results and discussion

PT is classified according to the aetiology, mechanism (i.e. which muscles are responsible) and the radiological features (the presence of inferior olivary hypertrophy or not) [1,2,3]. In 1949, Stern et al. suggested that PT was the human homolog of a primitive accessory respiratory reflex in gill-breathing vertebrates [5]. EPT can be triggered by central, nasopharyngeal, psychogenic and peripheral stimulation [3,4] On the other hand, SPT occurs due to a lesion in the dentato-rubral-olivary pathway, manifesting as the hypertrophic olivary degeneration of the medulla.

PAPT is characterised by the progression of ataxia in patients with PT. Some authors reported PAPT as a disorder of undetermined/degenerative aetiology and not due to an identifiable brain stem lesion [6,7]. However, the delayed and progressive worsening of cerebellar function associated with SPT secondary to the identified structural lesion has also been reported in the literature [8]. These patients could equally be classified as having a PAPT syndrome with an identifiable lesion [9]. Sporadic PAPT can similarly be classified as either idiopathic or essential [10].

It is preferable to consider PAPT as a third category of PT, instead of considering it to be a subtype of SPT to avoid inconsistency and confusion. However, difficulty with the classification is likely to persist unless post-mortem histopathology or advanced imaging modalities become widely available.

## Pathogenesis of essential palatal tremor (EPT)

### I. Central origin

No clear structural pathology is found on magnetic resonance imaging (MRI) in patients with EPT but a functional MRI revealed potential generators of tremor in the inferior olive and brainstem [11]. Alhough this observation pointed towards a potential single generator for both EPT and SPT, the final pathway for the two tremors is different [1,2,3]. EPT is triggered by the contraction of the tensor veli palatini (TVP) muscle. The TVP is supplied by the trigeminal (5th cranial) nerve [2]. Another observation to support the central origin of the EPT is the response of said tremors to centrally-acting drugs like lamotrigine, sodium valproate and falunarizine [12,13,14]. However, these were presented through anecdotal evidence and no placebo-controlled trial was ever conducted to assess the true response.

### II. Peripheral origin

The inflammation of the oral and nasal mucosa was found to be common among the patients with EPT. It was reported that EPT could be triggered by an upper respiratory tract infection [3,15]. EPT was also reported to have been cured after an adenoidectomy and tonsillectomy [3]. EPT can also be influenced by pressure changes in the ear canal and by the changes in the tone and position of the pharyngeal muscles [16,17]. However, such continuous movements cannot be maintained for prolonged periods in the absence of a central generator. The ear click is an important clinical feature of the EPT [1,2,3,12,13,14]. If a peripheral inflammation is the sole trigger for ear click, then it should appear with the tremor [18]. However, a peripheral insult can act as a potential trigger of the central tremor generator [3,4].

### III. Voluntary

Some people can voluntarily contract the TVP, the main muscle involved in EPT. Klein et al. reported a family where the members could voluntarily produce a palatal tremor without any structural pathology of the peripheral or central nervous system [19]. Such people have probably acquired control over the central tremor generator part of the brain. These voluntary movements can be seen in tic disorders or psychogenic movement disorder, although prolonged maintenance of the rhythmical movements is rare in tics [3].

### IV. Psychogenic

A psychogenic origin of EPT has been reported [20,21]. Unlike voluntary EPT, patients with psychogenic EPT are more distressed by such movements. The common triggers for psychogenic origin are emotional stress or previous trauma. Many such patients demonstrated classic clinical signs of psychogenic disorders like distractibility, entertainability and variability. However, sometimes these clinical features can also be seen in non-psychogenic EPT, making the distinction difficult [21,22]. Many such patients have no obvious psychiatric co-morbidities or other psychogenic movement disorders [3,22].

## Pathogenesis of symptomatic palatal tremor (SPT)

**Figure 1:**
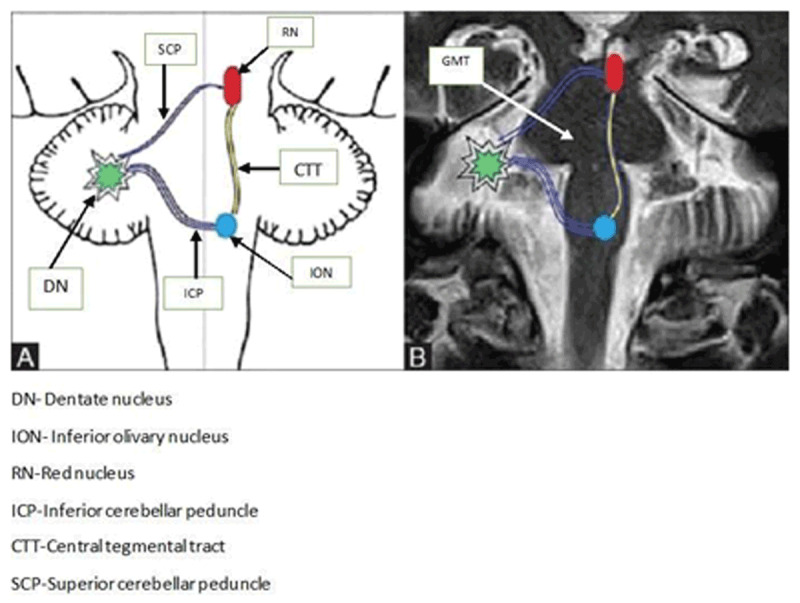
The Guillain-Mollaret Triangle (1A-schematic in the left and coronal Magnetic resonance imaging on the right-1B) -formed by the Red Nucleus (RN), Dentate Nucleus (DN) of the Cerebellum, Inferior Olivary Nucleus (ION), SCP – Superior Cerebellar Peduncle (connects the DN to the RN), ICP – Inferior Cerebellar Peduncle (connects the DN to the ION), CTT – Central Tegmental Tract (connects RN to the ION).

SPT is triggered by the contraction of the levator veli palatini muscle (LVP). The LVP is supplied by the 9th and 10%^th^ cranial nerves. SPT arises from a lesion within the Guillain Mollaret triangle (GMT) (Figure 1) [1,10,23,24,25,26,27]. The GMT is a conceptual triangle in the brainstem. The GMT has three corners i.e. the red nucleus (RN) of the ipsilateral midbrain, the inferior olivary nucleus (ION) of the ipsilateral medulla and the dentate nucleus of the contralateral cerebellum [1,25,26,27]. The central tegmental tract CTT) connects the RN to the ION. The dentatorubral tract connects the RN to the contralateral dentate nucleus via the superior cerebellar peduncle (SCP). The inferior cerebellar peduncle (ICP) joins the ION to the contralateral dentate nucleus (DN) [1,23,24,25,26,27,28].

ION hypertrophy appears after 4–6 months of the initial insult [24,25,26,27,28]. The ION undergoes trans-synaptic degeneration characterised by the hypertrophy of the ION [24,25,28,29]. This hypertrophic olivary degeneration (HOD) represents the vacuolation of the ION and the enlargement of the cell bodies resulting from a lesion of the GMT [24,26,27,28]. When the primary lesion is in the dentato-rubral tract of SCP, the HOD is contralateral. However, bilateral HOD happens when both the CTT and SCP are involved [24].

The DN is connected to the contralateral ION by the GABA (gamma amino butyric acid) -ergic inhibitory projections which in turn send the excitatory projections to the Purkinje cells of the cerebellum [27]. The neurons within the ION are interconnected by the gap junctions and they can act as an asynchronised neuronal ensemble in normal circumstances [1]. These oscillations work as pacemakers in timely processing, temporal co-ordination, and cerebellar motor learning. The denervated olivary neurons released from the inhibitory inputs enlarge and develop sustained synchronised oscillations leading to the palatal tremor. The appearance of SPT may depend on the hyperactivity of the olivary neurons released from inhibitory inputs until the peak of both IOH and SPT is reached [25,26]. Nishie et al. postulated that the persistence of peak intensity and the distribution of SPT is likely due to both the disturbance of the natural rhythmicity of the body and the lack of feedback from the abnormal movement resulting from the dysfunction of the olive [29]. SPT appears after a median of 10–11 months following the primary lesion [10,28]. SPT reaches a peak between 5–24 months after the lesion [28,29].

Recently, Shaikh et al. proposed a dual oscillator model for the pathogenesis of oculopalatal tremor (OPT) [30].Oculopalatal tremor refers to the synchronous combination of PT and pendular nystagmus. In this interesting model, the interaction between an oscillator in the inferior olivary nucleus and a modulator in the cerebellum was proposed to be the trigger for OPT. The cerebellar cortex was proposed to be an amplifier of the ocular tremor without changing its frequency. However, Kattah et al. recently reported the observation of OPT without any HOD [31]. The maladaptive cerebellar plasticity can trigger an oculopalatal tremor even when the inferior olive is not the primary source of oscillation [31].

## Pathogenesis of progressive ataxia and palatal tremor (PAPT)

There are limited studies available on the origin of the PAPT. Cilia et al. noticed an impairment of the dentato-rubro-olivary pathway and the nigrostriatal dopamine system in patients with idiopathic PAPT [32]. The authors concluded that the RN was involved in the pathophysiology of idiopathic PAPT. The inflammation of the GMT was postulated in PAPT [25,33]. Recently, Mari et al. found an insoluble four-repeat (4R) tau deposition in the inferior olive of the medulla [34]. The authors hypothesised that this tau deposition could trigger the retrograde degeneration of the dentato-olivary fibers. This retrograde degeneration might cause secondary (deafferentation type) hypertrophic degeneration in other olivary neurons potentially through the loss of axon collaterals. Recently, more widespread 3R (repeat) and 4R tau deposition has been reported in the postmortem study of two patients with PAPT [7]. The current histopathological association of the sporadic PAPT with tau deposition points towards a primary neurodegenerative process [7,28].

## Causes of palatal tremor: (Table 1) [7,8,9,10,23,27,28,35,36,37,38,39,40,41,42,43,44,45,46,47,48,49,50,51,52,53,54,55,56,57,58,59,60,61,62,63,64,65,66,67,68]

EPT is idiopathic. SPT can occur due to any lesion in the dentato-rubro-olivary pathway of the GMT [1,23,24,25,26,27,28]. The common causes of SPT are infarct (hemorrhagic or ischemic), tumour, vascular malformation, drugs and demyelination (Table 1) [24,25,26,27,28]. PAPT can be familial or sporadic [7,10,28,32,33,62,63,64,65,66,67,68]. The familial causes of PAPT are Alexander’s disease, polymerase gamma gene (POLG) mutation, and spinocerebellar ataxia type 20 [28,58,59,60,61,62,63,64,65,66,67]. Sporadic PAPT is likely to be neurodegenerative in origin.

**Table 1:** Causes of palatal tremor.

Symptomatic palatal tremor

**I) Vascular/Ischemic:**
Basilar artery occlusion [36] Brainstem or cerebellar infarct/bleed/tumor Vertebral artery dolichectasia [37] Vascular malformation (Arteriovenous malformation, aneurysm) [24,25,26,27,28,38]
**II) Inflammatory/Demyelinating:**
Multiple Sclerosis [39,40] Neurosarcoidosis [41] Multifocal Leukoencephalopathy Bechet’s disease [42]
**III) Genetic:**
Spinocerebellar ataxia [18,43]
**IV) Infectious:**
Tick-borne meningoencephalitis/Listeria encephalitis [44,45] Whipple disease [46]
**V) Neoplastic:**
Intestinal lymphoma [47] Posterior fossa tumour
**VI) Neurodegenerative:**
Progressive supranuclear palsy [48]
**VI) Miscellaneous:**
Traumatic brain injury [49] Amyotrophic Lateral Sclerosis [50] Drugs (Ciprofloxacin/Lithium/Carbamazepine) [51,52] Epilepsy [53] Hashimoto encephalopathy [54,55] Anti -GAD antibodies mediated encephalitis [56]
**Progressive Ataxia and Palatal Tremor (PAPT)**

**I) Sporadic:**
Tauopathy [7] Gluten sensitive ataxia [33] Vascular malformation [57]
**II) Familial/genetic:**
Alexander disease [58,59] Spinocerebellar Ataxia –20 (SCA 20) [60,61] POLG mutation [62,63] Hereditary spastic paraparesis type 7 (HSP-7) [64,65] GM2 Gangliosidosis [66] Neuroferritinopathy [67] Cerebrotendinous Xanthomatosis [68]

## Clinical features (Table 2)

### I) Essential Palatal tremor (EPT)

**Table 2:** Additional clinical features of various subtypes of palatal tremor.

Essential Palatal Tremor

Audible ear clicking [3,23]
**Symptomatic Palatal Tremor [6,23,24,25,26,27,28]**

Holmes tremor Oromandibular tremor Myoclonus Ataxia Nystagmus Dysarthria Dysphagia Throat clicking Ophthalmoplegia Optic atrophy Ocular tremor Facial myokymia Epilepsy partialis continua Intranuclear ophthalmoplegia
**Progressive Ataxia and Palatal Tremor (PAPT) [7,10,28,58,59,60,61,62,63,64,65,66,67]**

Pendular nystagmus/oscillopsia Hypermetric and hypometric saccades Ophthalmoplegia Optic atrophy (SPG 7, POLG mutation) Movement disorders (chorea, dystonia, parkinsonism, myoclonus – SPG 7, SCA 20, Neuroferritinopathy, POLG mutation) Pyramidal tract signs (hemiparesis, paraparesis – SCA 20, SPG 7 mutation) Neuropathy (SPG 7, POLG) Cognitive impairment (POLG mutation, Alexander disease, Neuroferritinopathy) Seizure (POLG mutation, Alexander disease) Hearing loss (mainly in sporadic PAPT) Muscle disorders (POLG mutation) Dysarthria Dysphonia (SCA 20) Dysphagia Dysmorphic feature (GM2 Gangliosidosis) Macrocephaly (Alexander disease) Diarrhea (celiac disease, GM2 Gangliosidosis) Hypogonadism, stroke like episodes (POLG mutation)

**Abbreviations:** EPT: Essential palatal tremor. SPT: Symptomatic palatal tremor. PAPT: Progressive ataxia and palatal tremor. SPG 7: genetic mutation responsible for hereditary spastic paraparesis. SCA 20: Spinocerebellar ataxia 20. POLG: Polymerase gamma gene.

Although TVP is the main muscle involved in the generation of the EPT, the contraction of other muscles like the oropharyngeal muscles, masseter and temporalis were also reported [28].

Zadikoff et al. reviewed the existing literature on EPT and found a male: female ratio of 1:1 [3]. Deuschl et al. reported the persistence of EPT during sleep in 50% of patients [3].The frequency of EPT is not only highly variable among different patients but it can also vary within a single individual [3,28].

An audible ear click was reported very commonly with EPT [3,23,28]. The ear click is produced by the contractions of the TVP muscle that opens the eustachian tube, causing a sudden decrease in the surface tension within the tube [3,23]. The ear click can be audible or inaudible, unilateral or bilateral. Some people have learned to elicit or regulate the frequency and volume of EPT [19]. The ear-clicking has been described as ticking, banging, cracking, popping, clattering, crunching or crackling noises [3]. Sometimes the ear click can be very distressing. EPT can show distractibility and entrainment during the clinical examination, which is suggestive of a functional or psychogenic element. Although sensory tricks like pressing over the mastoid or adopting certain neck positions were reported to reduce the tremor, such manoeuvres probably worked by altering the position and tone of the muscles involved or by changing the pressure in the ear canal [3].

### II) Symptomatic palatal tremor (Table 2)

Since SPT happens due to a lesion in the GMT, many clinical features can be seen other than palatal tremor [23,24,25,26,27,28]. Common associated clinical features reported were ophthalmoplegia, oculopalatal tremor, rubral or Holmes tremor, dysarthria, dysphagia and ataxia. Since SPT is commonly found in patients with an ischemic or hemorrhagic infarct, pyramidal tract signs can also be seen. Pendular nystagmus can be seen in up to 30% of cases with SPT [28]. The rarer clinical features reported were optic atrophy, vertical gaze paresis, facial dyskinesia, seizure and encephalopathy. Ophthalmoplegia was observed more frequently in unilateral HOD while ocular myoclonus and generalised myoclonus was observed in patients with bilateral HOD [27]. Ear clicking is rare in SPT [28]. However, tremors can also be observed in other derivatives of the branchial arch like the larynx, pharynx or diaphragm [28]. PAPT shows the relentless progression of ataxia. Sporadic PAPT reveals abnormal eye findings (saccadic pursuit, pendular nystagmus), oscillopsia and progressive ataxia

EPT can be distinguished from the SPT by the presence of an audible click, the variability of the tremors, the disappearance of tremors during sleep (50% cases), a lack of other clinical signs (ataxia, tremor and ophthalmoplegia) and a lack of any structural lesions (HOD) in MRI brain in EPT.

Table 3 summarises the important differences between EPT and SPT (pathogenesis, muscles involved, clinical features and investigations etc).

**Table 3:** The differences among the various subtypes of palatal tremor.

	Essential Palatal Tremor (EPT)	Symptomatic Palatal Tremor (SPT)	Progressive ataxia with Palatal Tremor (PAPT)

Main muscle involved in the generation of tremor [3,23]	Tensor veli palatini	Levator veli palatini	Levator veli palatini
Nerve supply of the main muscle [3,23]	5%^th^ cranial nerve	9%^th^ and 10%^th^ cranial nerves	9%^th^ and 10%^th^ cranial nerve
Abnormal reflex types [23]	Polysynaptic	Monosynaptic, oligosynaptic and polysynaptic	Not known
Relationship with sleep [23]	Disappears in 50% cases	Persists	Persists
Effect of anaesthesia on tremor	disappears	persists	Not known
Auditory signs	Audible ear click -Common	Audible ear click-rare	Tinnitus, sensorineural hearing loss Audible ear click – rare (familial)
Ocular signs	Rare	Can be seen	Torsional and horizontal nystagmus, INO, hypermetric saccades, reduced VOR, vertical gaze palsy, optic atrophy
Other clinical features	Rare except the ear click, entrainment common	Ataxia, tremor, dysarthria etc – not entrainable	Familial PAPT – additional pyramidal tract signs like tetraparesis, progressive ataxia, chorea, dystonia, cognitive impairment, autonomic dysfunction, tendon xanthoma Sporadic PAPT: dysarthria, dysphagia
Cause	Unknown	Sporadic -any Lesion (infarct, bleed, tumour etc) within the Guillain Mollaret triangle of brainstem or idiopathic/neurodegenerative	Familial/genetic – (POLG mutation, Alexander disease, Celiac disease, Cereberotendinous Xathomatosis, Celiac disease, GM2 Gangliosidosis) Neuroferritinopathy Sporadic-Neurodegenerative/tauopathy/MSA/Gluten sensitive
Magnetic resonance imaging of brain	No structural deficit	Hypertrophic olivary degeneration of medulla	Familial -significant brainstem atrophy but no HOD, dark dentate nucleus, cerebellar atrophy, iron accumulation in basal ganglia (Neuroferritinopathy) White matter lesion with frontal predominance (Alexander disease) Sporadic PAPT-HOD

## Investigations (Table 4)

The diagnosis of EPT is mainly clinical but investigations can be helpful in SPT. Routine blood tests are usually less informative although some specific blood tests like Lyme serology, celiac screening, Angiotensin-converting enzyme (ACE; for neurosarcoidosis), cholestanol and bile alcohol (cerebrotendinous xanthomatosis), anti-GAD antibodies (glutamatic acid decarboxylase; GAD encephalitis) and anti-thyroid peroxidase (TPO) antibodies (Hashimoto’s encephalopathy) can be helpful to identify the causes of SPT in the right clinical context [33,40,41,55,56,68]. The cerebrospinal fluid study can be helpful to rule out malignancy (lymphoma) or to help in the clinical diagnosis of multiple sclerosis (presence of an unmatched oligoclonal band), neurosarcoidosis (ACE) and Whipple’s disease. Similarly, genetic testing for spinocerebellar ataxia (SCA 18 & 20), hereditary spastic paraparesis type 7 (caused by *SPG 7* gene mutation), Alexander’s disease (glial fibrillary acid protein) and *POLG* mutation (mitochondrial disorders) can be helpful in reference to familial PAPT [43,58,60,61,63,64,65]. A surface electromyogram (EMG) can be useful to measure the frequency of the palatal tremor. Similarly, in some patients with PAPT (SCA20, HSP7 and mitochondrial disorders), a nerve conduction study can reveal axonal neuropathy.

**Figure 2:**
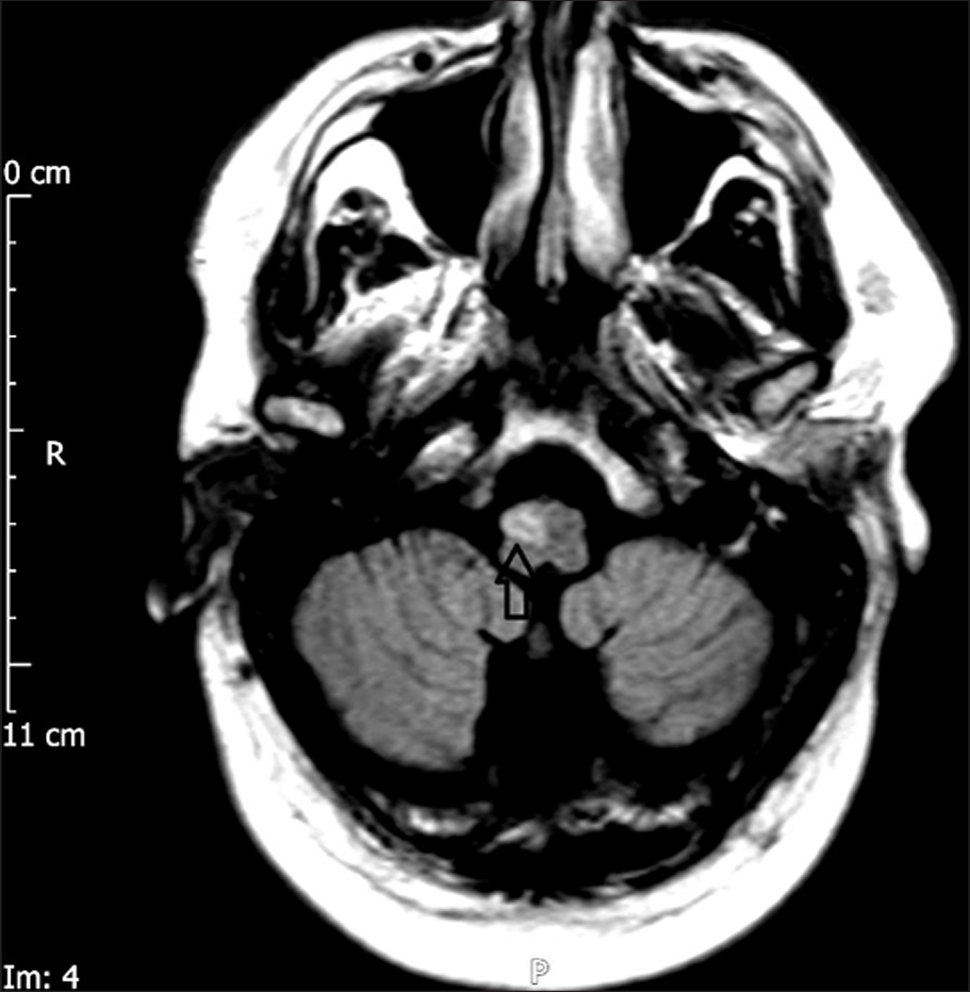
Axial T2 Weighted Fluid Attenuated Inversion Recovery (FLAIR) Magnetic Resonance Imaging (MRI) shows Increased Signal of the Inferior Olivary nucleus of the Right Hemi-medulla (black arrow) Suggestive of the Hypertrophic Olivary Degeneration.

The most characteristic finding shown on the MRI of the head of patients with SPT and sporadic PAPT is the HOD of the medulla [10,11,23,24,25,26,27,28,29,30,32,33,35,69]. As mentioned before, the HOD is a transsynaptic degeneration of the inferior olivary nucleus of the medulla because of a lesion within the boundary of the GM triangle [24,25,26,69]. The HOD can be unilateral or bilateral (Figure 2) [24,25,26]. A bilateral HOD is more likely to be idiopathic [25,26,27]. Four different patterns of HOD were described in the literature based on the location of the primary lesion. They were an ipsilateral HOD with a primary lesion in the brainstem, a contralateral HOD with a primary lesion in the cerebellum/cerebellar peduncle, a bilateral HOD with both CTT affected by a midline lesion and a bilateral HOD with the primary lesion in the cerebellum or unilateral brainstem [26].

The familial variant of PAPT shows brainstem and cervical cord atrophy but no HOD [10,68,70]. Sporadic PAPT shows mostly bilateral HOD. One study revealed hemosiderin in the T2 gradient Echo MRI brain in sporadic PAPT due to superficial hemosiderosis [57]. The same study found a brainstem vascular malformation in 3 patients. When Alexander’s disease causes PAPT in adults, the ‘tadpole’ pattern of brainstem atrophy can be found [71].

Goyal et al. detailed three distinct phases of the MRI corresponding to the pathological changes of the HOD [69]. The first stage is the increased T2 signal in the ION without hypertrophy after 4–6 months of the initial insult. The second stage is the increased T2 signal and ION hypertrophy that resolves in 3–4 years after the lesion. During the third stage, the olivary hypertrophy starts to disappear. The atrophy begins after a few years and the olivary shrinkage becomes apparent. However, the increased T2 signal of the MRI may persist. Recently, diffusor tensor imaging (DTI), an advanced MRI technique, has been able to show the specific disruption in the various components of the GM triangle [72]. The DTI revealed probable demyelination in the late stages of the HOD.

**Figure 3:**
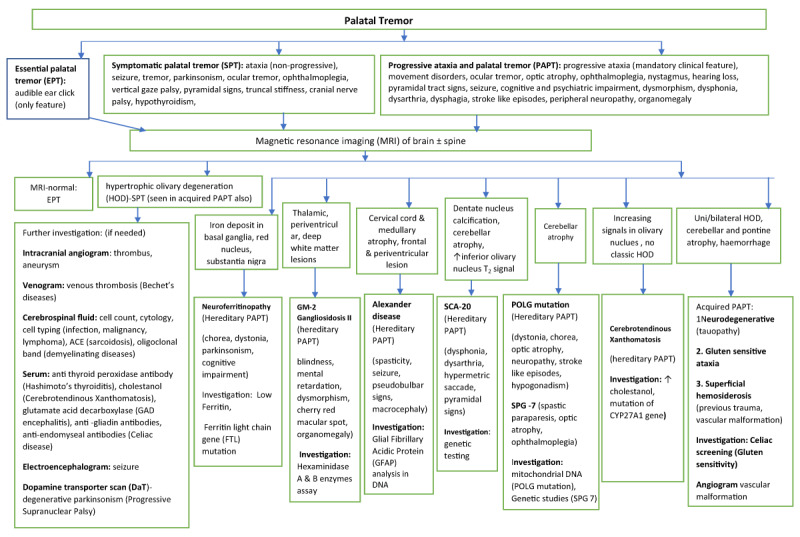
Algorithmic Approach to the Investigation of Palatal Tremor.

Figure 3 provides as algorithmic approach to investigating the various subtypes of palatal tremor.

## Treatment

The literature is sparse so far as the treatment of palatal tremor is concerned. EPT was reported to be responsive to clonazepam, carbamazepine, phenytoin, valproate, gabapentin, flunarizine, lamotrigine, trihexyphenidyl, sumatriptan and botulinum toxin [73]. Pandurangi et al. reported an 80% improvement in ear click and a significant improvement of the essential palatal tremor with clonazepam (up to 3 mg/day) [74]. Borggreve et al. reported the complete resolution of essential palatal tremor within a few days of valproate intake [13]. Cakmur et al. reported the complete resolution of EPT after starting on flunarizine and the same patient only had a partial response to valproate before [14]. Scott et al. reported the abolition of essential palatal tremor after the administration of oral and subcutaneous sumatriptan [75]. However, another case report found no response to sumatriptan [76]. Jabbari et al. noticed that there was a good response to the anticholinergic Trihexyphenidyl [77]. However, Fabini et al. reported no improvement of EPT with valproate, carbamazepine, levodopa in one patient with EPT but the resolution of EPT with high dose clonazepam (12 mg/day) in another patient [78]. One case report observed the slowing down of the essential palatal tremor with lamotrigine (125 mg twice daily) [12]. Piracetam was found to be effective in childhood onset EPT though the tremor recurred when piracetam was stopped [79]. Cognitive behavioural therapy can also be helpful [80]. The injection of botulinum toxin into the TVP can help to reduce the tremor and ear clicking noise [81,82]. Low doses (e.g. onabotulinumtoxin A – 4 to 30 U mainly) were injected under EMG guidance to treat the symptomatic ear click [81,82]. The botulinum toxin was found to be more effective in the paediatric age group [83]. Radiofrequency ablation can also be effective at abolishing a tremor of the soft palate [84].

A palatal tremor usually does not bother the patient although any associated oscillopsia or ataxia can be disabling. Gabapentin and memantine could be helpful to reduce the oscillopsia in an oculopalatal tremor [28,85]. The treatment of SPT involves the treatment of the associated medical cause (if possible) or the surgical removal of any underlying lesion. Cheung et al. reported the resolution of ciprofloxacin-induced PT with valproate [51]. However, it is difficult to gauge to what extent the discontinuation of Ciprofloxacin contributed to the resolution of the tremor. Iwasaki et al. reported a significant improvement of palatal myoclonus (tremor) in a patient with Bechet’s disease through the use of ceruletide, a cholecystokinin-like peptide [42]. The botulinum toxin injection was effective at reducing symptomatic PT in tick-borne meningoencephlitis [45]. Salazar et al. reported on the prophylactic role of levetiracetam at eliminating the risk of palatal tremor after a cortical ischemic infarct of the brain [86]. Marnane et al. reported new-onset epilepsy with palatal tremors due to the anti-glutamic acid decarboxylase antibodies that responded to intravenous immunoglobulin [56].

There is no known therapy to halt the progression of ataxia in PAPT although ataxia in one patient improved with a gluten-free diet [33]. Rossi et al. reported a mild improvement of gait instability but not the palatal tremor after treatment with chenodeoxycholic acid in a patient with PAPT secondary to cerebrotendinous xanthomatosis [68]. Nasal sumatriptan 20 mg temporarily helped palatal tremor although lamotrigine and clonazepam were found to be ineffective [70]. Drugs that can reduce the electronic coupling among the hypetrophic olivary neurons by blocking the connections (quinine or mefloquine) are suggested to control the symptoms although no study is available to support this [28].

Surgery had a limited role in the treatment of palatal tremor (except in the removal of any underlying lesion in the GMT). The surgical perforation of the tympanic membrane and the excision of the LVP, TVP and tensor tympani muscle did not provide any obvious benefit [82]. A thalamotomy also did not provide benefit in a patient with both PT and Holmes’ tremor [87]. The bilateral deep brain stimulation of the red nucleus did not reduce OPT in one patient [28]. However, one group reported the resolution of palatal tremor with no recurrence for 5 years after stereotactic ablative surgery involving the zona incerta of the right subthalamus and the nucleus ventralis intermedius of the right thalamus (VIM) [38].

## Conclusion and future directions

Whether the term ‘isolated palatal tremor’ instead of the ‘essential palatal tremor’ would better represent the varied aetiologies or not needs to be settled. PAPT should be classified as a separate category of PT to maintain a consistency in the descriptions in the literature and to better facilitate future research. Further studies are also needed to understand the relationship between mitochondrial disorders and HOD. This is as mitochondrial disorders are found to have caused PAPT. Similarly, the relationship between tau inclusions (3 repeat or 4 repeat) and sporadic PAPT needs to be explored further. Combined clinical and autopsy studies would be more helpful to find the actual causes of HOD as many patients with bilateral HOD show no clear underlying aetiology. The more widespread use of Diffusor Tensor Imaging (DTI) and magnetic resonance imaging in the future would throw more light on the pathophysiology of palatal tremors. Although PT usually does not bother the patients, ataxia, nystagmus or oscillopsia can be disabling. Future studies are needed to explore new therapeutic approaches as the current management for the aforementioned symptoms is unsatisfactory.
